# Comparison of fast acquisition strategies in whole‐heart four‐dimensional flow cardiac MR: Two‐center, 1.5 Tesla, phantom and in vivo validation study

**DOI:** 10.1002/jmri.25746

**Published:** 2017-05-04

**Authors:** Pankaj Garg, Jos J.M. Westenberg, Pieter J. van den Boogaard, Peter P. Swoboda, Rahoz Aziz, James R.J. Foley, Graham J. Fent, F.G.J. Tyl, L. Coratella, Mohammed S.M. ElBaz, R.J. van der Geest, David M. Higgins, John P. Greenwood, Sven Plein

**Affiliations:** ^1^ Leeds Institute of Cardiovascular and Metabolic Medicine (LICAMM) University of Leeds Leeds United Kingdom; ^2^ Leiden University Medical Center Leiden The Netherlands; ^3^ Philips Healthcare Guildford United Kingdom

**Keywords:** 4D flow cardiac MR, phase‐contrast magnetic resonance imaging, MR flow imaging, flow quantification, validation

## Abstract

**Purpose:**

To validate three widely‐used acceleration methods in four‐dimensional (4D) flow cardiac MR; segmented 4D‐spoiled‐gradient‐echo (4D‐SPGR), 4D‐echo‐planar‐imaging (4D‐EPI), and 4D‐*k‐t* Broad‐use Linear Acquisition Speed‐up Technique (4D‐*k‐t* BLAST).

**Materials and Methods:**

Acceleration methods were investigated in static/pulsatile phantoms and 25 volunteers on 1.5 Tesla MR systems. In phantoms, flow was quantified by 2D phase‐contrast (PC), the three 4D flow methods and the time‐beaker flow measurements. The later was used as the reference method. Peak velocity and flow assessment was done by means of all sequences. For peak velocity assessment 2D PC was used as the reference method. For flow assessment, consistency between mitral inflow and aortic outflow was investigated for all pulse‐sequences. Visual grading of image quality/artifacts was performed on a four‐point‐scale (0 = no artifacts; 3 = nonevaluable).

**Results:**

For the pulsatile phantom experiments, the mean error for 2D PC = 1.0 ± 1.1%, 4D‐SPGR = 4.9 ± 1.3%, 4D‐EPI = 7.6 ± 1.3% and 4D‐*k‐t* BLAST = 4.4 ± 1.9%. In vivo, acquisition time was shortest for 4D‐EPI (4D‐EPI = 8 ± 2 min versus 4D‐SPGR = 9 ± 3 min, *P* < 0.05 and 4D‐*k‐t* BLAST = 9 ± 3 min, *P* = 0.29). 4D‐EPI and 4D‐*k‐t* BLAST had minimal artifacts, while for 4D‐SPGR, 40% of aortic valve/mitral valve (AV/MV) assessments scored 3 (nonevaluable). Peak velocity assessment using 4D‐EPI demonstrated best correlation to 2D PC (AV:r = 0.78, *P* < 0.001; MV:r = 0.71, *P* < 0.001). Coefficient of variability (CV) for net forward flow (NFF) volume was least for 4D‐EPI (7%) (2D PC:11%, 4D‐SPGR: 29%, 4D‐*k‐t* BLAST: 30%, respectively).

**Conclusion:**

In phantom, all 4D flow techniques demonstrated mean error of less than 8%. 4D‐EPI demonstrated the least susceptibility to artifacts, good image quality, modest agreement with the current reference standard for peak intra‐cardiac velocities and the highest consistency of intra‐cardiac flow quantifications.

**Level of Evidence**: 1

**Technical Efficacy**: Stage 2

J. Magn. Reson. Imaging 2018;47:272–281.

Four‐dimensional flow cardiac MR (4D flow cardiac MR) is increasingly used in clinical and research applications for complex aortic and intra‐cardiac flow assessment. 4D flow cardiac MR is a 3D phase‐contrast magnetic resonance imaging (PC MRI) method with 3D velocity encoding allowing post hoc time‐resolved 3D visualization and retrospective quantification of blood flow at any location in a 3D volume. 4D flow cardiac MR enables a wide variety of options for visualization and quantification of intra‐cardiac flow, ranging from basic aspects such as flow volume and peak velocity to more complex analyses such as the estimation of hemodynamic effects at the vessel wall and myocardium, as well as visualization of flow pathways in the heart and great vessels.^1^ Integrating 4D flow cardiac MR in routine clinical protocols has been challenging, in part due to long scan times.

Several data acceleration methods have been used to shorten scan times in 4D flow cardiac MR, including radial under‐sampling, parallel imaging, *k‐t* Broad‐use Linear Acquisition Speed‐up Technique (BLAST), *k‐t* Sensitivity encoding (SENSE), generalized auto‐calibrating partially parallel acquisitions (GRAPPA), echo‐planar imaging (EPI), Iterative self‐consistent parallel imaging reconstruction (L1‐SPIRiT), and 5‐point PC‐vastly undersampled isotropic voxel radial projection imaging (VIPR).^2^, ^3^, ^4^, ^5^, ^6^, ^7^, ^8^, ^9^

With the use of some of these accelerated acquisition methods free‐breathing “whole‐heart” 4D flow cardiac MR can be performed in a total scan time of 10 min or less depending on size of the heart, heart rate and patient habitus.^7^, ^10^, ^11^ A recently published consensus document recommends the use of 4D segmented fast spoiled gradient echo (4D‐SPGR) pulse sequences for 4D flow cardiac MR with a segmentation factor of 2 based on the experience of research institutions.^1^ However, validation of this recommended technique versus other published acceleration techniques for whole‐heart acquisition in clinical applications is needed for both clinical and research applications.

Therefore, the aim of the present study was to (1) validate three commonly applied acceleration methods in whole‐heart 4D flow cardiac MR: 4D‐SPGR with k‐space segmentation, nonsegmented 4D echo‐planar imaging (4D‐EPI), and 4D‐*k‐t* BLAST (4D‐*k‐t* BLAST) in static and pulsatile flow phantoms, and (2) investigate these three acceleration methods in vivo in healthy volunteers for quality, consistency, and reliability to quantify intra‐cardiac flow velocity and volumes.

## Materials and Methods

The study was conducted across two centers and was approved by the ethics committees at both. The study complied with the Declaration of Helsinki. All volunteers gave written informed consent.

### 4D Flow Cardiac MR Studies

Comprehensive information on the details of pulse‐sequences and corrections for all the 2D and 4D flow acceleration methods are included in the online Supplementary Document S1, which is available online.

### Ex vivo: Static and Pulsatile Phantom Experiments

Detailed information on the flow phantom setup in the MR system and the experiments are included in the online Supplementary Document S1.

### In Vivo: 4D Flow Cardiac MR Studies

Twenty‐five (n = 25, 14 at Leeds, 11 at Leiden) healthy adult volunteers were recruited for the in vivo study. Exclusion criteria included: history of cardiovascular disease and any contraindication to cardiac MR imaging. All volunteers underwent cardiac MR imaging on identical 1.5 Tesla (T) systems at both sites (Ingenia, Philips, Best, The Netherlands); with a 28‐channel flexible torso coil, with digitization of the MR signal in the receiver coil. The cardiac MR protocol included baseline survey, cines (vertical long axis, horizontal long axis, short‐axis contiguous left‐ventricle volume stack), 2D phase contrast acquisition through the aortic and mitral valve, which was then followed by all the three 4D flow accelerated methods, which were done in arbitrary order. These 4D flow methods are detailed in the online Supplementary Document S1.

### Image Analysis

Image analysis of phantom data as well as in vivo cines, 2D PC flow quantification, and 4D flow quantification was performed offline using MASS software (Version 2016EXP, Leiden University Medical Center, Leiden, The Netherlands). For both static as well as pulsatile phantom measurements, an a priori circular‐shaped luminal region of interest (ROI) was defined, static for all phases, with an area size imposed by the phantom specifications (i.e., 0.79 cm^2^). This ROI was manually placed on the first phase of a time series and copied to subsequent phases. For all phantom 4D flow data sets, image analysis was performed for the three center slices and measurements were averaged among the three samples to reduce the effect of noise.

**Figure 1:**
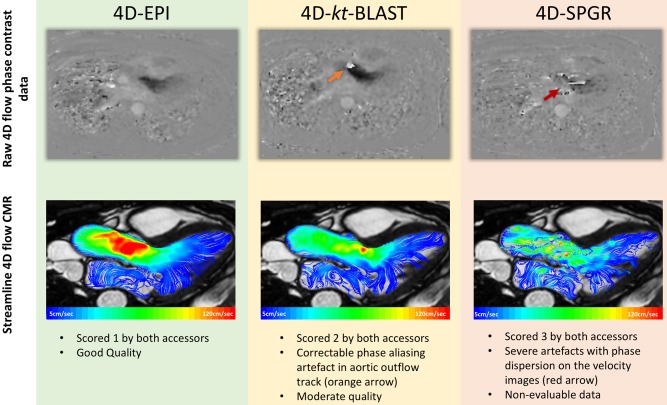
Scoring of image artifacts was done on the raw 4D flow data for each directional phase contrast data (as demonstrated in first row). Case example: In this case, there is velocity aliasing artifact for 4D*‐k‐t* BLAST acquisition (orange arrow) (all acquisitions at velocity encoding = 150 cm/s). This velocity aliasing artifact can be corrected and does not limit quantification. 4D‐SPGR had severe phase dispersion artifact which limited quantification. Streamlines demonstrate better velocity profile for 4D‐EPI versus 4D‐SPGR (second row).

In vivo left ventricular volumes and the ejection fraction (EF) were derived from cine images using standard methods.^12^ For each 4D flow cardiac MR pulse sequence, blinded quality checks were performed by two experts from the two centers: JW; a cardiac MR academic with over 15 years' experience and PG; a cardiac MR academic with over 3 years' experience (Fig. 1). From the 4D flow data, phase contrast and magnitude images were interrogated for the following types of artifacts: signal void, distortions, phase wrap and phase dispersion. Visual grading of image quality and presence of artifacts was done on a 4‐point scale: 0; excellent quality with no artifacts present, 1; good quality but with some blurring artifacts on magnitude images but none on velocity images, 2; moderate quality with substantial blurring on both magnitude and velocity images, 3; severe artifacts with phase dispersion on the velocity images present in the region of interest, leading to nonevaluable data.

### Flow and Velocity Assessment

In volunteers, all 4D flow assessments were done using validated techniques including retrospective valve tracking, with measurement planes positioned perpendicular to the inflow direction on two‐ and four‐chamber cines.^13^ Background velocity correction (i.e., for correction of through‐plane motion and phase offset) was used from the velocity sampled in the myocardium and phase unwrapping was performed on source images when aliasing occurred in the area of interest as per previously published guidelines on phase‐contrast methods.^14^ Contour segmentation was performed manually.

Peak velocities at aortic valve (AV) (peak systolic) and mitral valve (MV) (early filling phase) were obtained from reformatted planes with identical orientation and position as the static 2D PC MR planes. To investigate the reliability of peak velocity derived by all 4D flow methods, 2D PC MR was used as reference method. Valvular net forward flow (NFF) at the MV and the AV were calculated using all methods. Consistency of flow assessment for all methods was investigated by comparing mitral inflow and aortic outflow NFF.

### Statistical Analysis

Statistical analysis was performed using IBM SPSS^®^ Statistics 21.0. Continuous variables are expressed as mean ± SD. Normality for quantitative data was established using the Shapiro‐Wilk test. Demographic comparisons were performed with an independent samples t‐test. For image quality check, paired Wilcoxon test was used to establish any significant changes. For paired comparison student t‐test was used. Additionally, agreement between two methods was expressed as bias according to the Bland‐Altman analysis and by coefficient of variability (CV). Correlation assessment between two parameters was done using the Pearson's correlations (r). All statistical tests were two‐tailed; *P* values < 0.05 were considered significant. For investigating agreement between mitral and aortic stroke volumes of the three 4D flow acquisitions, we performed repeated measures analysis of variance with Bonferroni correction.

## Results

### Phantom Experiments

The mean error in flow volume assessment (compared with time beaker measurements) for the 2D PC sequence was −0.8 ± 1.2% (*P* = 0.22) for the static experiments and −1.0 ± 1.1% (*P* = 0.001) for the pulsatile experiments. For static experiments, the mean error for 4D−SPGR was −5.2 ± 1.1% (*P* = 0.003), for 4D−EPI −7.1 ± 1.9% (*P* = 0.001), and for 4D‐*k‐t* BLAST −4.9 ± 3.8% (*P* = 0.03). For the pulsatile experiments, the mean error for 4D‐SPGR was −4.9 ± 1.3% (*P* = 0.002), for 4D‐EPI −7.6 ± 1.3% (*P* < 0.001) and for 4D‐*k‐t* BLAST −4.4 ± 1.9% (*P* = 0.008). For these measurements, the mean error (compared with the 2D PC acquisition) in peak velocity assessment for 4D−SPGR was −3.2 ± 1.2% (*P* < 0.001), for 4D−EPI −5.3 ± 2.2% (*P* = 0.01) and for 4D‐*k‐t* BLAST −8.8 ± 2.3% (*P* < 0.001).

### In Vivo Experiments

Baseline volunteer demographics and cardiac MR parameters are detailed in Table 1. Seventeen (68%) of the volunteers were male and the mean age of all volunteers was 38 ± 14years. There were no significant differences in characteristics between volunteers from the two centers.

**Table 1:** Healthy Volunteer Demographics and Baseline Cardiac MR Scan Parameters*

	All (n = 25)	Center 1 (n = 14)	Center 2 (n = 11)	*P*‐Value
Age, years	38 ± 15	38 ± 15	39 ± 15	0.61
Male	17(68%)	10 (71%)	7 (64%)	0.69
Weight, kg	76 ± 13	77 ± 14	76 ± 12	0.83
Height, cm	171 ± 8	173 ± 7	169 ± 8	0.15
Heart rate, bpm	63 ± 9	63 ± 10	63 ± 9	0.94
LVEDV_i_, mL/m^2^	90 ± 17	91 ± 2	91 ± 12	1
LVESV_i_, mL/m^2^	34 ± 11	35 ± 13	33 ± 8	0.66
SV_i_, mL/m^2^	56 ± 8	55 ± 8	57 ± 8	0.55
LV Mass_i_, g	54 ± 11	54 ± 12	53 ± 9	0.88
Ejection fraction, %	63 ± 6	62 ± 7	63 ± 5	0.62
Mitral regurgitation fraction, % (number of volunteers)	1.6 ± 1.4 (18)	2 ± 2 (11)	1 ± 1(7)	0.17
Aortic regurgitation fraction, % (number of volunteers)	0.6 ± 1 (9)	0.9 (7)	1.1 (2)	0.20

*Values are mean ± SD. LV measurements are indexed to body surface area (BSA).

LVEDV_i_ = left ventricular end diastolic volume (indexed); LVESV_i_ = left ventricular end systolic volume (indexed); SV = stroke volume.

### Acquisition Time and Image Quality Assessments

**Figure 2:**
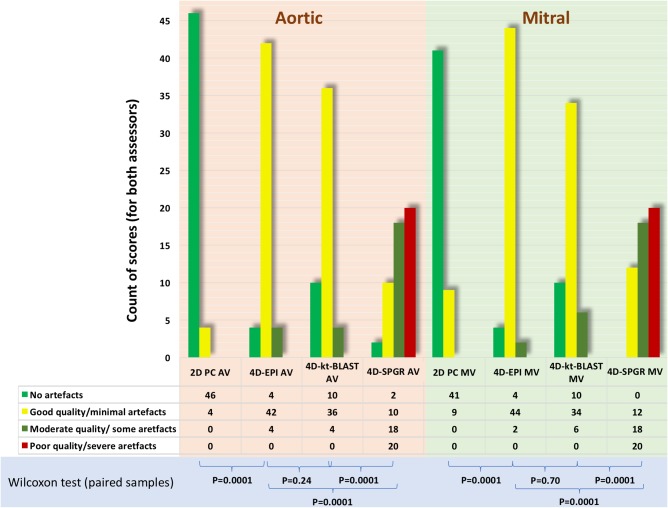
Image quality of mitral and aortic valve flow acquisitions using the different acceleration techniques. Both for the aortic and mitral valve, 2D PC image quality was the best. 4D‐EPI and 4D‐*k‐t* BLAST did not differ much in image quality. 4D‐SPGR was poorest in image quality.

The acquisition time was shortest for 4D‐EPI, and it was statistically significantly shorter than for 4D‐SPGR (8 ± 2 min versus 9 ± 3 min, *P* < 0.01). Even though the acquisition time for 4D‐*k‐t* BLAST was not statistically different to 4D‐EPI (8 ± 2 min versus 9 ± 3 min; *P* = 0.29), it was on average almost 1 min longer. Agreement between two assessors, who were blinded to each other's analysis for artifact scoring demonstrated good reliability (kappa = 0.95, 95%CI 0.92–0.98). Artifact scoring for images across mitral and aortic flows was similar for 4D‐EPI and 4D‐*k‐t* BLAST (MV; *P* = 0.43, AV; *P* = 0.16) (Figs. 1 and 2). However, 4D‐SPGR acquisition was of inferior quality when compared with 4D‐EPI (MV; *P* < 0.001, AV; *P* < 0.001) and 4D‐*k‐t* BLAST (MV; *P* < 0.001, AV; *P* < 0.001). Additionally, 10(40%) 4D‐SPGR mitral and aortic flows were of the lowest quality grade of 3 rendering their analysis not feasible.

### Intra‐cardiac Peak Velocity Assessment

#### Mitral Valve Inflow

Peak MV early‐diastolic inflow velocity (E) by 4D‐EPI demonstrated good correlation to 2D PC acquisition (r = 0.71; *P* = 0.001), whereas 4D‐SPGR derived E‐velocity had modest correlation (r = 0.59; *P* = 0.01). 4D‐*k‐t* BLAST derived E‐velocity demonstrated the poorest correlation (r = 0.42; *P* = 0.03).

#### Aortic Valve Outflow

Peak systolic AV outflow velocity by 4D‐EPI demonstrated best correlation to 2D PC acquisition method (r = 0.78; *P* < 0.001). 4D‐SPGR and 4D‐*k‐t* BLAST demonstrated modest correlation to 2D PC acquisition (4D‐SPGR: r = 0.56, *P* = 0.04; 4D‐*k‐t* BLAST: r = 0.59, *P* = 0.002) (Table 2).

**Table 2:** Consistency and Variability of Mitral Inflow and Aortic Outflow Measurements Between All Acquisitions (Aortic Versus Mitral)

	Mean	SD	Pearson's correlation	Bias (95% CI)	Paired Student t‐test*	CV (%)
**Net** f**orward** f**low (mL)**						
2D PC AV	95	18	0.83, *P* < 0.001	−9 (−14 to −4)	<0.01	11
2D PC MV	104	20				
4D‐SPGR AV	85	16	0.58, *P* = 0.02	−5 (−14 to 4)	0.28	29
4D‐SPGR MV	89	20				
4D‐EPI AV	91	18	0.94, *P* < 0.001	−2 (−5 to 1)	0.14	7
4D‐EPI MV	93	17				
4D‐*k‐t* BLAST AV	82	15	0.57, *P* = 0.003	12 (6 to 18)	<0.01	20
4D‐*k‐t* BLAST MV	70	17				
**Peak velocity (cm/s)**						
2D PC AV	128	20	…	…	…	…
2D PC MV	82	17	…	…	…	…
4D‐SPGR AV	152	24	0.56, *P* = 0.04	−22 (−31 to 12)	<0.001	21
4D‐SPGR MV	98	35	0.59, *P* = 0.01			
4D‐EPI AV	131	22	0.78, *P* < 0.001	−2 (−7 to 2)	0.21	10
4D‐EPI MV	84	22	0.71, *P* < 0.001			
4D‐*k‐t* BLAST AV	118	22	0.59, *P* = 0.002	10 (5 to 15)	<0.001	14
4D‐*k‐t* BLAST MV	73	14	0.42, *P* = 0.03			

*P‐value.

**Figure 3:**
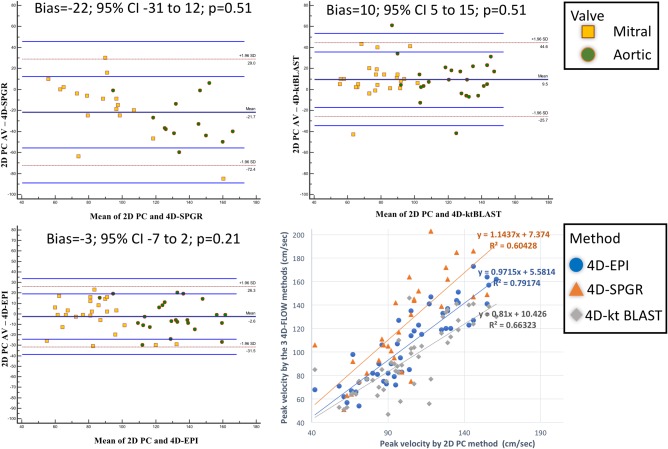
Bland Altman analysis and scatter plots for the assessment of peak velocity using all the acceleration methods. 4D‐SPGR had maximum bias (‐22, 95% CI: ‐31–12) versus 2D PC acquisition. 4D‐EPI demonstrated least bias (‐3, 95% CI: ‐7–2) and highest correlation (R^2^ = 0.79) to 2D PC acquisition for peak velocity assessments.

Peak velocity assessment for both MV and AV by 4D‐EPI demonstrated the best correlation to the reference 2D PC acquisition (4D‐EPI: r = 0.89, *P* < 0.001; 4D‐SPGR: r = 0.78, *P* < 0.001; 4D‐*k‐t* BLAST: r = 0.81, *P* < 0.001) (Fig. 3). The overall bias for peak velocity assessment was lowest for 4D‐EPI (−2 cm/s, 95%CI −7 to 2 cm/s; *P* = 0.21) with a CV = 9.8%. The other acquisition methods demonstrated significant bias and CV (4D‐*k‐t* BLAST: bias = 10 cm/s, 95%CI 5 to 15 cm/s; *P* < 0.001 and CV = 14.3%, 4D‐SPGR: bias = ‐22 cm/s, 95%CI −31 to 12 cm/s; *P* < 0.001 and CV = 21.4%) (Table 2; Fig. 3).

### Consistency of Flow Volume Assessment

**Figure 4:**
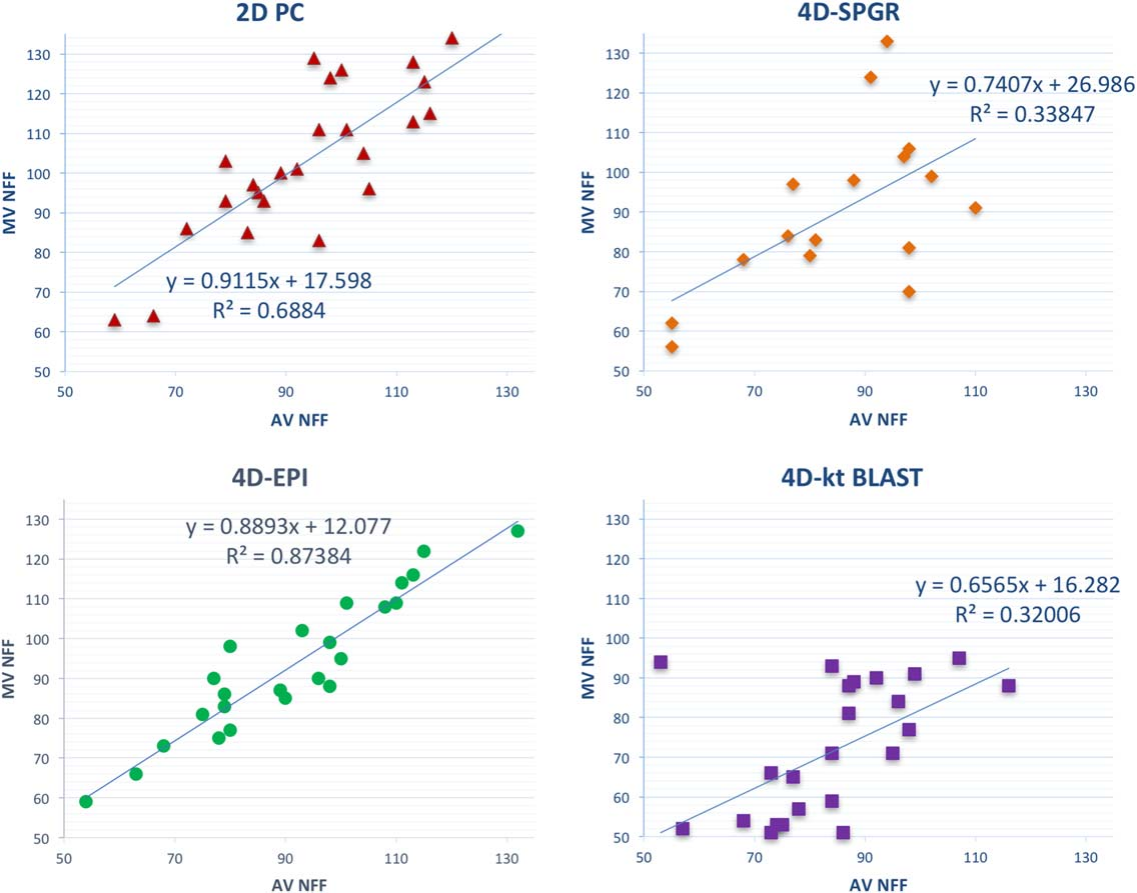
Scatter plots of net forward flow (NFF) correlation through the mitral and aortic valve to investigate consistency between all the four methods. 4D‐EPI demonstrated the best consistency (R^2^ = 0.87) versus 4D‐*k‐t* BLAST acquisition which demonstrated least correlation (R^2^ = 0.32) between NFF of MV and AV.

NFF (or effective stroke volume) through the MV and AV correlated best for 4D‐EPI with lower correlation for the other 4D acquisition methods and 2D PC (4D‐EPI: r = 0.94, *P* < 0.001 versus 2D PC: r = 0.83, *P* < 0.001 versus 4D‐SPGR: r = 0.58, *P* = 0.02 versus 4D‐*k‐t* BLAST: r = 0.57, *P* = 0.003) (Fig. 4). Bias for NFF between MV and AV was significant for 2D PC (bias = −9 mL, 95%CI –14 to −4 mL; *P* < 0.01) and 4D‐*k‐t* BLAST (bias = 12 mL, 95%CI 6 to 18 mL; *P* = 0.01) acquisitions (Table 2). Between MV and AV, bias and CV for the NFF were lowest for the 4D‐EPI (bias = 2 mL, 95%CI –5 to 1 mL; *P* = 0.135; CV = 7%;). The CVs were considerably high for the 4D‐*k‐t* BLAST (20%) and the 4D‐SPGR (29%) acquisitions.

The mean difference between MV and AV NFF was significantly different for 4D‐*k‐t* BLAST versus 4D‐EPI (*P* = 0.0007) or 4D‐SPGR (*P* = 0.002) (Supplementary Fig. 2).

### Diastolic Flow Assessment

Assessment of diastolic function parameters from the transmitral flow‐time curves using 2D PC acquisition with static plane differed significantly to retrospective valve tracking assessment for all the three 4D flow acquisitions (Table 3). The mean peak E velocity was significantly higher for 4D‐EPI than for 2D PC acquisition (90 ± 21 cm/s versus 82 ± 17 cm/s; *P* = 0.03). The mean peak E velocity was lowest for 4D‐*k‐t* BLAST at 73 ± 13 cm/s.

**Table 3:** Diastolic Flow Assessment Using All Methods[Link]

	Mean	SD	Paired Student t‐test compared to 2D PC	Paired Student t‐test compared to 4D‐EPI
**2D phase contrast**				
E, cm/s	82	17	…	…
A, cm/s	49	10	…	…
E volume, mL	78	18	…	…
A volume, mL	25	7	…	…
E/A	1.8	0.6	…	…
PEFR, mL/s	603	151	…	…
PLFR, mL/s	301	77	…	…
**4D‐SPGR**				
E, cm/sec	112	26	0.000	<0.001
A, cm/sec	69	24	0.003	<0.001
E volume, mL	68	17	0.004	0.549
A volume, mL	22	7	0.051	0.321
E/A	1.8	0.5	0.725	0.052
PEFR, mL/s	495	127	0.000	0.368
PLFR, mL/s	277	72	0.148	0.253
**4D‐EPI**				
E, cm/s	90	21	0.035	…
A, cm/s	48	12	0.861	…
E volume, mL	70	15	0.012	…
A volume, mL	22	6	0.012	…
E/A	1.9	0.6	0.057	…
PEFR, mL/sec	508	99	0.001	…
PLFR, mL/sec	270	80	0.043	…
**4D‐*k‐t* BLAST** a				
E, cm/s	73	13	0.041	<0.001
E volume, mL	67	17	0.007	0.366
PEFR, mL/s	415	117	<0.001	<0.001

*2D PC acquisition was assessed in static plane and the 4D flow techniques were assessed using retrospective valve tracking for flow quantifications.

a Late mitral filling parameters (A‐wave) cannot be quantified for 4D‐*k‐t* BLAST because of prospective ECG gating.

PEFR = peak early flow rate; PLFR = peak late flow rate.

There was a trend toward a difference in E/A ratio between 2D PC versus 4D‐EPI; *P* = 0.06 and 4D‐EPI versus 4D‐SPGR, *P* = 0.05. However, this was not the case for 2D PC versus 4D‐SPGR (*P* = 0.72).

## Discussion

The present study, involving in vitro and in vivo experiments to investigate robustness, accuracy, and applicability of acceleration methods of 4D flow cardiac MR for whole‐heart imaging and valvular flow assessment demonstrated the following: (1) in phantom models, all 4D flow acceleration techniques demonstrated mean error of less than 8% compared with in vitro flow and velocity assessment; (2) in vivo, 4D‐EPI requires the shortest acquisition time versus segmented 4D‐SPGRand 4D*‐k‐t* BLAST acceleration, and 4D‐EPI had the best image scores; (3) additionally, 4D‐EPI demonstrated the best correlation and least bias for intra‐cardiac peak velocities compared with the reference method of 2D PC acquisition; and, (4) finally, 4D‐EPI demonstrated the best agreement between aortic valve NFF and mitral valve NFF and, therefore, showed highest internal consistency.

4D flow cardiac MR has evolved in recent years as a clinical application for evaluating intra‐cardiac flow. The use of modern data acceleration methods to speed up data acquisition in particular has allowed a wider application of this technique in clinical research studies. Several studies have investigated acceleration methods for flow assessments.^13^, ^15^, ^16^, ^17^, ^18^, ^19^, ^20^, ^21^, ^22^, ^23^ These studies have not only used different acceleration methods, but also different flow‐encoding schemes (symmetric, asymmetric, Hadamard 4‐point encoding, 5‐point‐encoding or multipoint encoding), different field‐strengths (mainly 1.5T/3T systems), different scan parameters (field of view, spatio‐temporal resolution) and not necessarily with intra‐cardiac assessment for quality and consistency of flow. Vendor‐specific acceleration techniques have been investigated by several research groups to investigate consistency and reliability of intra‐cardiac flow assessment using 4D flow cardiac MR. One of these studies used GRAPPA (20), three studies used EPI,^13^, ^18^, ^23^ two studies L1‐SPIRiT,^15^, ^17^ two studies compared *k‐t* BLAST acceleration and SPGR (SENSE) acceleration,^7^, ^24^ and one study used SPGR (SENSE).^25^

We compared the current recommended 4D‐SPGR (with twofold parallel imaging, SENSE) approach with two other acceleration techniques, EPI and *k‐t* BLAST, which have also been widely used in clinical research studies implementing whole‐heart 4D flow cardiac MR.^1^ In vitro validation showed that 2D PC was very accurate with respect to flow volume assessment, presenting an error of 1%. All 4D flow cardiac MR assessments underestimated flow volume, with mean error lower than 8%. Even though, 4D‐EPI had shortest acquisition time, this was clinically comparable to both 4D‐SPGR and 4D‐*k‐t* BLAST.

Our in vivo results showed, as expected, substantial bias for internal flow consistency between AV and MV flow 2D PC cardiac MR, as the acquisition plane in this approach remains fixed during the acquisition and MV flow volume is overestimated compared with AV flow. This is in agreement with previously published results.^13^, ^18^ Furthermore, internal consistency was poor for 4D‐*k‐t* BLAST, as this acquisition uses prospective triggering and as a result, part of diastole is missing resulting in an underestimation of MV flow volume. In line with previously published results,^13^, ^18^ internal consistency was high for 4D‐EPI in our in vivo studies. 4D‐SPGR showed more variation and less correlation, and therefore, had a poorer overall performance than 4D‐EPI. Also direct assessment of peak velocity for 4D‐EPI was in good agreement with 2D PC, while the other two techniques showed substantial bias, moderate agreement and higher variation. 4D‐EPI scored slightly below 4D‐SPGR and 4D‐*k‐t* BLAST in the in vitro experiments. However, 4D‐EPI had a better performance in vivo, both for reliability in flow mapping and with respect to the presence of artifacts. A possible explanation for this might be the reduction of motion‐related artifacts due to the fast EPI k‐space filling strategy. In the current study, respiratory motion compensation was not used in vivo as this saves considerable acquisition time. However, in the consensus statement,^1^ the use of 4D‐SPGR is recommended with respiratory motion compensation. Hence, the performance of 4D‐SPGR might have improved considerably if navigator gating was implemented.

Initial studies using 4D‐*k‐t* BLAST demonstrated underestimation of flow because of time blurring issues with prospective gating.^24^ Zaman et al demonstrated that using contemporary coil systems, this can be mitigated and reliable peak velocity and stroke volume assessment can be made using respiratory navigated 4D‐*k‐t* BLAST acquisition.^7^ However, our study findings differ, and are more in line with the work by Carlsson et al who showed that 4D‐*k‐t* BLAST demonstrated underestimation of flow in vivo.^24^ Additionally, because of the use of prospective ECG triggering and the consequent lack of data for the full cardiac cycle, 4D‐*k‐t* BLAST could not estimate peak late diastolic mitral inflow (A) velocity. The work by Zaman et al was at 3.0T and used respiratory navigation for all their acceleration techniques, however, the present study did not. Hence, the underestimation of flow velocities and poor CV for the 4D‐*k‐t* BLAST acquisition in the present study may be due to the combination of lower signal to noise at 1.5T and inherent temporal blurring from prospective triggering and the respiratory motion. Additionally, Zaman et al did not investigate mitral inflow which may have an important role for intra‐cardiac diastolic and multi‐parametric 4D flow assessments. It would be plausible to suggesst that without respiratory navigation and at 1.5T, 4D‐*k‐t* BLAST is not suitable for intra‐cardiac flow quantification.

More recently, Petersson et al demonstrated that retrospectively gated intra‐cardiac 4D flow MRI can be sped up without the use of acceleration techniques using respiratory navigated spiral trajectories.^26^ This method does not compromise in spatio‐temporal windows and demonstrates reliability and consistency for intra‐cardiac flow quantification including diastolic inflow indices. However, mean scan times for spiral acquisition in their study were higher (13 ± 3 min) than the most reliable acceleration technique in the present study, 4D‐EPI (8 ± 2 min), most likely because Petersson used respiratory navigation. Moreover, the present study, which was done without respiratory navigation, demonstrated similar robust linear correlation between NFF through the mitral and aortic valves (this study: R^2^ = 0.87 versus Petersson study: R^2^ = 0.90).

There were several limitations to our study. The in vitro validation of the various 2D and 4D flow acquisition techniques was performed using a straight tube with a laminar flow, creating idealized flow conditions. The use of a more realistic heart phantom model would provide more complex intra‐cardiac flow patterns that would probably give more insight in the performance of the various cardiac MR flow acquisition techniques under more physiological conditions.

Additionally, even though we tried to keep all acquisitions parameters similar for the three 4D flow methods, occasionally, one method would need slight adjustment of the number of reconstructed phases. In case a volunteer had a high heart rate, the temporal acquisition window of the sequence did not allow reconstruction of 30 phases for all sequences and then the highest number of reconstructed phases possible was chosen. This study was limited to one vendor and 1.5 Tesla MR system. Respiratory navigation was omitted in this study which could have influenced and resulted in differences in the results of in vivo experiments. However, respiratory navigated 4D flow acquisitions substantially increase acquisition time and hence are less clinically applicable in real‐world. For intra‐cardiac flow quantification, Kanski et al showed that respiratory motion compensation does not have significant effect in healthy volunteers.^25^ Finally, no patients were recruited to this study as it would be unethical to put patients through long 4D flow cardiac MR acquisitions.

In conclusion, of the three 4D flow cardiac MR methods tested at 1.5T field‐strength, 4D‐EPI demonstrated the least susceptibility to artifacts, good image quality, modest agreement with the current reference standard for peak intra‐cardiac velocities and the highest consistency of intra‐cardiac flow quantifications.

## Supporting information

Additional supporting information may be found in the online version of this article

Supporting Information
