# Supplementary material for: Comparison of fast acquisition strategies in whole‐heart four‐dimensional flow cardiac MR: Two‐center, 1.5 Tesla, phantom and in vivo validation study
Source: J Magn Reson Imaging. 2017 May 4;47(1):272–81. doi: 10.1002/jmri.25746 (PMC5801550; doi:10.1002/jmri.25746)
Supplement: Supplementary file 1 — Supporting Information [file JMRI-47-272-s001.docx]

**Supplementary Document**

# 4D flow cardiac MR studies

4D flow cardiac MR refers to time-resolved three-dimensional volume acquisition with spoiled gradient-echo schemes designed for interleaved velocity encoding in all three directions. Simple four-point velocity encoding was performed by the traditional “MPS” (measurement, phase, slice) encoding which implies sequential encoding of velocity in three directions and with respect to the frame of reference, which is the three-dimensional volume (1). The three velocity sensitized acquisitions are obtained after the first velocity compensated acquisition. Short echo times (TE) and repetition times (TR) are used to enable optimal temporal resolution. In this study, three different 4D flow cardiac MR sequences were used. These specific sequences were chosen as they are implemented on the available MRI platform and all three are already widely used for whole-heart 4D flow cardiac MR. Net acquisition times for each of the 4D flow pulse sequences were recorded and typical acquisition times of these three sequences are comparable (i.e., between 8-10 minutes) for free-breathing, non-respiratory-motion compensated whole-heart 4D flow cardiac MR. The major difference between these sequences is the choice of k-space filling acceleration: k-space segmentation, EPI read-out and k-t BLAST. Volume properties (field-of-view, acquisition voxel size, number of reconstructed slices) of the acquisition were kept identical among the three sequences. In vivo acquisition voxel size was kept as close as possible to 3×3×3 mm^3^. Field-of-view and number of slices (i.e., the 3D volume) was adapted to the subject’s size. TE and TR were set to shortest and the number of reconstructed phases was set to 30. If scanning or reconstructing with this set number of phases was not possible due to a high heart rate, the highest number of reconstructed phases possible was selected. Originally, the flip angle was set to 10º for all three sequences. When heart rate was high, requiring the number of reconstructed phases to drop below 20 (which happens occasionally for 4D-*kt* BLAST), a flip angle of 5º was chosen in order to further shorten the temporal acquisition window. As per consensus statement (2), 4D-SPGR was performed with segmentation factor of 2. In case the number of phases lowered the segmentation factor to 1 (an effect of high heart rate), the highest number of reconstructed phases possible was chosen which was allowed for acquisition with segmentation factor of 2. The acceleration factors for 4D-EPI (i.e., EPI factor of 5) and 4D-*kt* BLAST (i.e., 5-fold acceleration with 11 lines of training data in both ky and kz direction) were chosen identical to published data (3, 4).

Respiratory motion was monitored continuously on the workstation by the radiographer performing the examination. Before 4D flow acquisitions, all healthy volunteers were requested to breath as consistently as possible throughout the three acquisitions. They were given clear instructions not to fall asleep.

# Ex-vivo: Static and pulsatile phantom experiments

Static and pulsatile flow phantom experiments were carried out using 48 ml diluted gadolinium based contrast agent (Dotarem, Guerbet, Gorinchem, The Netherlands) dissolved in 6 l water. The contrast agent had a concentration of 0.5 mmol/ml and relaxivity of 3.4 mmol^-1^.l.s^-1^, resulting in a theoretical T1 of 112 ms (5). A silicon tube with 1 cm luminal diameter was led through the wall of the MRI room towards the cardiac MR scanner. Static or pulsatile flow was applied from outside the MRI room, using a Sarns centrifugal pump which was connected to a Sarns Delphin power supply and a 3M Sarns control module (3M Health care, Borken, Germany) (SFig 1A). Approximately 3 meters supplying and 3 meter returning tube was required to lead the flow in and out of the MRI room. The tube was placed inside a water tank, submerged under static water and positioned in the scanner iso-center.

In the static phantom setup, six static flows ranging from 2.52 l/min to 6.50 l/min were applied. Cardiac triggering was physiologically simulated by the scanner software at 120 beats/minute and for all 4D flow cardiac MR and 2D PC cardiac MR sequences, 10 cardiac phases were reconstructed. Next, six pulsatile flow settings were applied, ranging from 2.25 l/min to 5.20 l/min with a frequency of 61 cycles/minute. Cardiac MR triggering was performed by an external transistor-transistor-logic (TTL) trigger signal sent from the pump control unit to the MRI computer.

In-plane spatial resolution for 2D PC and the three 4D flow pulse sequences for the in-vitro setup were equal (1.5×1.5 mm^2^). Through-plane resolution for 4D flow was 1.5 mm (i.e., isotropic data), and slice thickness for 2D PC was 8 mm. To minimize angulated flow, the tube was positioned straight in the scanner, parallel in foot-head direction with water flowing from foot to head. The 3D volumes of the 4D flow cardiac MR acquisitions were identically planned (SFig 1), covering 1.35 cm (i.e., 9 slices of 1.5 mm thickness each) of the tube, and with the 2D imaging plane of the 2D PC cardiac MR positioned in the center of the 4D flow cardiac MR volume (SFig 1B). For all acquisitions, only through-plane flow velocity was analysed.

Before and after each MRI acquisition, a time-beaker measurement was performed distal to the phantom, at the end of the returning tube (SFig 1A) where volumetric collection was performed for 30 seconds. The flow rate was determined by averaging both time-beaker measurements.

For both phantom data as well as in vivo data, gradient non-linearity correction and Maxwell correction were compensated by the cardiac MR scanner. Remaining phase offset background errors (e.g. due to eddy currents) were corrected by background subtraction sampled near the area of interest (4). For phantom experiments, background correction was performed from a background ROI (indicated by the yellow contour in SFig 1C) enclosing the luminal ROI (indicated by the red contour) and for in vivo experiments from ROI positioned in myocardium (6, 7).

**References**

1. Pelc NJ, Bernstein MA, Shimakawa A, Glover GH: Encoding strategies for three-direction phase-contrast MR imaging of flow. *J Magn Reson Imaging* 1991; 1:405–13.

2. Dyverfeldt P, Bissell M, Barker AJ, et al.: 4D flow cardiovascular magnetic resonance consensus statement. *J Cardiovasc Magn Reson* 2015; 17:72.

3. Zaman A, Motwani M, Oliver JJ, et al.: 3.0T, time-resolved, 3D flow-sensitive MR in the thoracic aorta: Impact of k-t BLAST acceleration using 8- versus 32-channel coil arrays. *J Magn Reson Imaging* 2015; 42:495–504.

4. Westenberg JJM, Roes SD, Ajmone Marsan N, et al.: Mitral valve and tricuspid valve blood flow: accurate quantification with 3D velocity-encoded MR imaging with retrospective valve tracking. *Radiology* 2008; 249:792–800.

5. Westenberg JJ, Wasser MN, van der Geest RJ, et al.: Scan optimization of gadolinium contrast-enhanced three-dimensional MRA of peripheral arteries with multiple bolus injections and in vitro validation of stenosis quantification. *Magn Reson Imaging* 1999; 17:47–57.

6. Garg P, Hassell ME, Ripley DP, et al.: Reliability and reproducibility of trans-valvular flow measurement by 4D flow magnetic resonance imaging in acute myocardial infarct patients: two centre study. In *J Cardiovasc Magn Reson*. *Volume 18*. BioMed Central; 2016(Suppl 1):P36.

7. Calkoen EE, Westenberg JJM, Kroft LJM, et al.: Characterization and quantification of dynamic eccentric regurgitation of the left atrioventricular valve after atrioventricular septal defect correction with 4D Flow cardiovascular magnetic resonance and retrospective valve tracking. *J Cardiovasc Magn Reson* 2015; 17:18.

**Table 1.** *Ex-vivo*, in phantom cardiac MR experiments: pulse sequence and post-processing details for all the four phase contrast acquisitions. Table adapted from 4D flow cardiac MR consensus document.

|  | Segmented 2D-SPGR | 4D-SPGR | 4D-EPI | 4D-*k-t* BLAST |
| --- | --- | --- | --- | --- |
| Acceleration method | Parallel imaging; sense factor 2.5 in phase-encode anterior-posterior (AP) direction and factor 1.5 in slice-select feet-head direction  Segmentation factor of 4 | Parallel imaging; sense factor 2 in phase-encode AP direction  Segmentation factor of 2 | Parallel imaging; sense factor 2 in phase-encode AP direction  Echo Planar Imaging EPI factor of 5 | 5-fold acceleration with 11 lines of training data in both the ky and kz directions |
| Flip-angle | 15 | 10 | 10 | 5-10 |
| VENC (cm/s) | 150 | 150 | 150 | 150 |
| FOV | 350 | 350 | 350 | 350 |
| TE | 4 | 3 | 5 | 2 |
| TR | 7 | 6 | 11 | 6 |
| Partial k-space coverage in phase- directions | 90% | 90% | 90% | 90% |
| Signal averages | 1 | 1 | 1 | 1 |
| ECG gating | Retrospective | Retrospective | Retrospective | Prospective |
| Number of slices | 1 | 9 | | |
| Acquired temporal resolution (msec) | 2×TR×segmentation factor = 56 | 4×TR×segmentation factor = 48 | 4×TR = 44 | 4×TR = 24 |
| Reconstructed number of phases | 30 | 25-30 | 30 | 15-30 |
| In-plane spatial resolution (acquired) | 1.5×1.5 | 1.5×1.5 | | |
| In-plane spatial resolution (reconstructed) | 0.7×0.7 | 1.5×1.5 | | |

**Table 2.** *In-vivo* cardiac MR studies: pulse sequence and post-processing details for all the four phase contrast acquisitions. Table adapted from 4D flow cardiac MR consensus document (2).

|  | 2D-PC | 4D flow cardiac MR | | |
| --- | --- | --- | --- | --- |
|  | Segmented 2D-SPGR | 4D-SPGR | 4D-EPI | 4D-*k-t* BLAST |
| Acceleration method | Parallel imaging; sense factor 2.5 in phase-encode anterior-posterior (AP) direction and factor 1.5 in slice-select feet-head direction  Segmentation factor of 4 | Parallel imaging; sense factor 2 in phase-encode AP direction  Segmentation factor of 2 | Parallel imaging; sense factor 2 in phase-encode AP direction  Echo Planar Imaging EPI factor of 5 | 5-fold acceleration with 11 lines of training data in both the ky and kz directions |
| Flip-angle | 10 º | 10 º | 10 º | 10 º |
| VENC (cm/s) | 150 | 150 | 150 | 150 |
| FOV | 350 | 350-400 | 350-400 | 350-400 |
| TE (ms) | 3 | 3 | 3.5 | 2 |
| TR (ms) | 5 | 5 | 10 | 4 |
| Partial k-space coverage in phase- encoding directions | 90% | 90% | 90% | 90% |
| Signal averages | 1 | 1 | 1 | 1 |
| ECG gating | Retrospective | Retrospective | Retrospective | Prospective |
| Respiratory compensation | Breath-hold acquisition | Free-breathing | | |
| Number of slices | 1 | 35-40 | | |
| Acquired temporal resolution (ms) | 2×TR×segmentation factor = 40 | 4×TR× segmentation factor = 40 | 4×TR = 40 | 4×TR = 16 |
| Reconstructed number of phases | 30 | 25-30 | 30 | 15-30 |
| In-plane spatial resolution (acquired) | 2.5×2.5 (slice thickness:8 mm) | 3×3×3mm (isotropic) | | |
| In-plane spatial resolution (reconstructed) | 1.2x1.2 (slice thickness:8 mm) | 2×2×3mm | | |

**SFig 1. Panel A:** Illustration to demonstrate static/pulsatile flow phantom setup. Flow of water was applied by a computer controlled pump. For pulsatile flow, ECG triggering was controlled by the pump connected with the MR computer. Flow of water was led through the wall to the MR machine. A tank filled with static water was positioned in the iso-center of the magnet. The tube was led through the tank. The returning tube was led out of the MR room through the wall. Distal to this returning tube, time beaker measurements were performed to calibrate flow volume. **Panel B:** Illustration to demonstrate the identical 3D volumes of the 4D flow cardiac MR acquisitions covering 1.35 cm (i.e., 9 slices of 1.5 mm thickness each) of the tube. The 2D imaging plane of the 2D PC cardiac MR positioned in the center of the 4D flow cardiac MR volume. For all acquisitions, only through-plane flow velocity was analysed. **Panel C:** Example of contour definition in the velocity encoded images of the phantom. The yellow contour surrounding the cross-sectional lumen of the tube is used for background correction. The red contour segmenting the lumen, is a predefined circular ROI with a fixed 0.79 cm^2^ area.

**
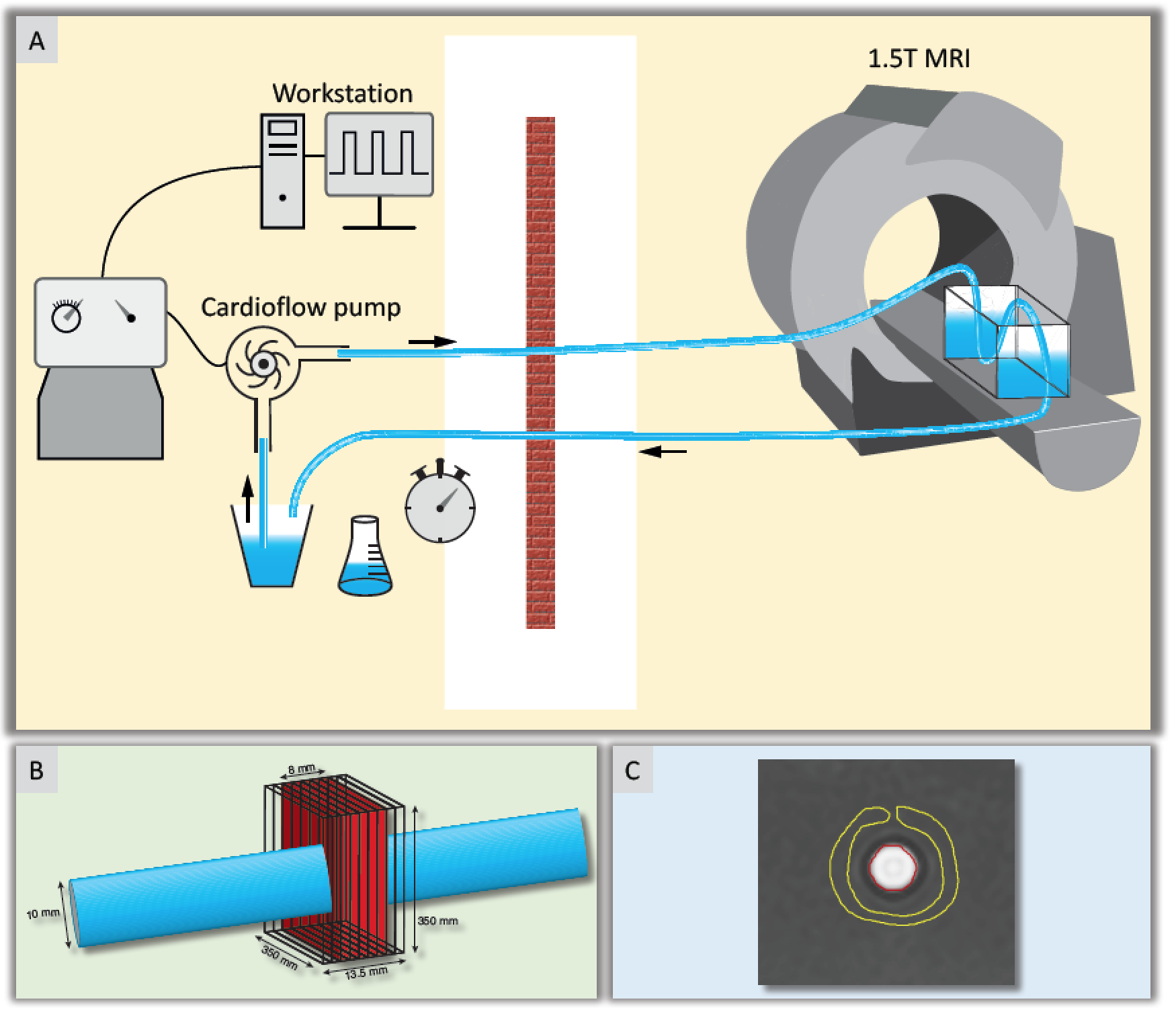
**

**SFig 2. Mean differences between mitral and aortic stroke volumes (SV) demonstrated that 4D-k-t-BLAST MV SV was consistently less than AV SV.**

**
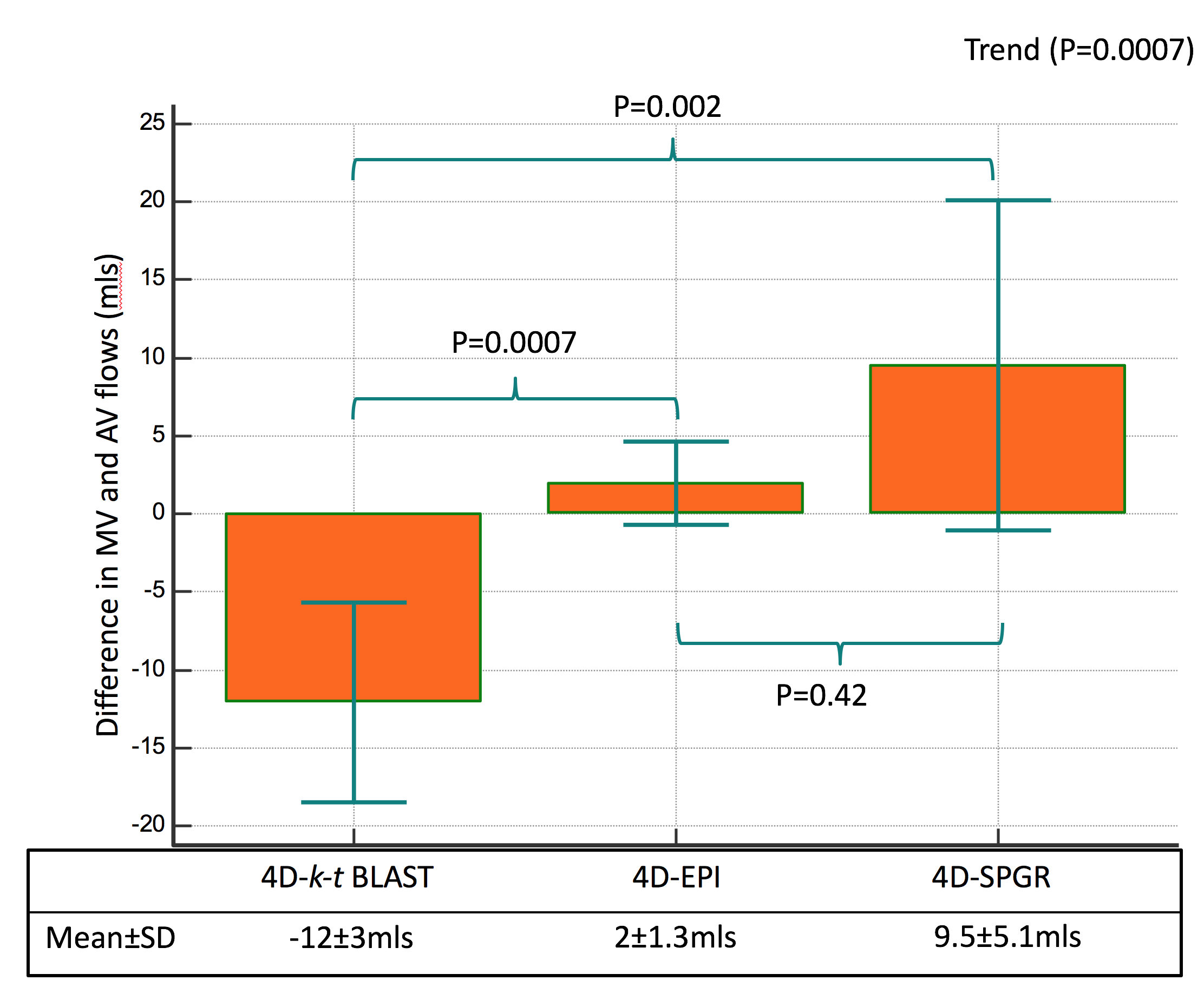
**
